# Editorial: Novel pharmacological interventions in emotional and neuropsychiatric disorders

**DOI:** 10.3389/fphar.2026.1974847

**Published:** 2026-09-28

**Authors:** Alba Garcia-Baos, Tamara Timic Stamenic, Jenny L. Fiedler, Snezana Kusljic

**Affiliations:** 1 University Bordeaux, CNRS, IMN, UMR 5293, Bordeaux, France; 2 Department of Anesthesiology, University of Colorado, Anschutz Medical Campus, Aurora, CO, United States; 3 Laboratory of Neuroplasticity and Neurogenetics, Department of Biochemistry and Molecular Biology, Faculty of Chemical and Pharmaceutical Sciences, Universidad de Chile, Santiago, Chile; 4 Department of Nursing School of Health Sciences, Faculty of Medicine, Dentistry and Health Sciences, The University of Melbourne, Parkville, VIC, Australia

**Keywords:** biomarkers, emotion, neurodevelopment, neuropsychiatric disorders, pharmacology

Emotional and neuropsychiatric disorders remain among the leading causes of disability worldwide (Santomauro et al., 2026), yet their therapeutic management continues to face substantial challenges. Although considerable progress has been made with currently available treatments, many patients experience incomplete symptom remission, delayed therapeutic responses, treatment failure, or adverse effects (Maalouf et al., 2011). These limitations reflect the biological complexity of psychiatric disorders, which arise from the interplay of neural circuitry, neurotransmission, immune responses, metabolism, genetics, and environmental influences (Gupta et al., 2026). Consequently, the field is undergoing a paradigm shift from symptom-oriented pharmacotherapy to mechanism-based interventions targeting specific biological processes that underlie disease vulnerability and progression. In this Research Topic, we present a Research Topic of original research articles and reviews by both basic science and clinical researchers that illustrate this transition and suggest a shift in the landscape of pharmacological interventions. It is therefore likely that future pharmacological interventions will emerge from integrating multiple levels of biological organization—from receptor signaling and neural circuits to immune regulation, the gut microbiome, and molecular biomarkers.

A recurring theme across the articles included in this Research Topic is the growing recognition that emotional and neuropsychiatric disorders arise from the dysregulation of interconnected biological systems rather than isolated neurotransmitter or neuroendocrine alterations. While serotonin, dopamine, and noradrenaline remain the central focus of current psychiatric treatments, increasing evidence indicates that developmental, genetic, and environmental factors dynamically shape their function. In this context, Palacios-Avedaño N et al. highlight that serotonergic function is profoundly influenced by developmental stage and biological sex, variables that have historically been overlooked in both preclinical and clinical research but are likely to contribute to differences in therapeutic efficacy. Consistent with this evolving view, novel receptor systems and neuromodulatory pathways that regulate emotional behavior have been identified beyond the classical monoaminergic hypothesis. Two original studies exemplified this concept through complementary investigations of cannabinoid-related signaling. Garcia-Gutierrez MS et al. demonstrated that loss of the non-canonical cannabinoid receptor GPR55 increased anxiety- and depression-like behaviors while altering amygdalar GABAAα2 receptor expression, highlighting a functional interaction between cannabinoid signaling and inhibitory neurotransmission in emotional regulation. Likewise, Zhu R et al. identified a circuit-specific mechanism through which endocannabinoid modulation influences depressive-like behaviors. Together, these findings reinforce the growing recognition that non-classical cannabinoid pathways constitute promising therapeutic targets (Cristino et al., 2019) and may provide new therapeutic avenues where conventional pharmacotherapies have shown limited efficacy. Zhang P et al. further emphasized the expanding complexity of receptor pharmacology. The authors discussed emerging evidence on sigma receptors as versatile modulators of neurotransmission, calcium homeostasis, synaptic plasticity, and neuroinflammatory responses, positioning them as attractive targets for next-generation antidepressant therapies. These contributions collectively underscore that understanding receptor biology requires considering cellular context, developmental timing, and individual biological variability if precision psychiatry is to become a clinical reality.

A second major theme emerging from this Research Topic is the central role of neuroimmune regulation. Neuroinflammation is now recognized as a common mechanism across numerous psychiatric and neurological disorders, influencing synaptic function, neurotransmitter release, neuronal survival, and, lastly, behavioral outcomes (Bhatia et al., 2025). Zhang Y et al. showed that inhibition of the RhoA/NF-κB signaling pathway attenuated hippocampal inflammation while improving behavioral manifestations of post-stroke depression, providing mechanistic support for targeting inflammatory cascades in affective disorders. Similarly, Oka N et al. reported that administration of the human placental extract ‘Melsmon’ suppresses neuroinflammatory responses in both central and peripheral tissues induced by a stress-sensitive mouse model of depression. Although the interventions differ pharmacologically, these studies converge on the idea that targeting neuroimmune dysfunction represents a promising strategy for developing more effective treatments. Rather than acting in isolation, neuroimmune signaling interacts extensively with neurotransmission and neural circuitry. For example, inflammatory mediators influence glutamatergic and GABAergic transmission, endocannabinoid signaling, neurogenesis, and synaptic remodeling (Martins and Harrison, 2026), linking several of the mechanisms explored throughout this Research Topic.

Beyond identifying novel therapeutic targets, another prominent feature of this Research Topic is the increasing integration of systems biology into neuropsychopharmacology. Traditional drug discovery has often relied on approaches focusing on single molecular targets. In contrast, several contributions to this Research Topic adopted multidimensional strategies that integrate metabolomics, microbiome profiling, and biomarker discovery. Du Y et al. combined metabolomic and gut microbiota analyses to explore the therapeutic actions of levo-tetrahydropalmatine against ketamine addiction, illustrating how systems-level approaches can identify coordinated biological responses. Complementing these findings, the review by Pan J et al. points out microbial-mediated mechanisms linking gut microbiota dysbiosis to stress-related psychopathology, emphasizing the microbiome as a promising therapeutic target. Extending this perspective further, Rosas-Sanchez GU et al. discussed the emerging concept of personalized psychobiotics, proposing that targeted modulation of the gut microbiota may influence emotional regulation through coordinated effects on neurotransmitter synthesis, neuroimmune signaling, and the gut-brain axis. Together, these studies illustrate that integrating metabolic, microbial, and molecular information can improve our understanding of disease mechanisms at the molecular level and also facilitate the identification of novel therapeutic targets and biomarkers that influence treatment response. Such multidimensional strategies are likely to become progressively important as the field moves toward precision and personalized medicine. The review of Ochando et al. further reinforces the concept of precision medicine by summarizing recent advances in identifying molecular biomarkers associated with neurodevelopmental alterations following prenatal alcohol exposure. The identification of reliable biomarkers capable of predicting long-term neurological vulnerability represents a critical step toward earlier intervention and individualized patient management. Such efforts complement mechanistic pharmacological research by providing tools to identify patient subpopulations most likely to benefit from targeted therapies.

The translational perspective represented within this Research Topic is equally noteworthy. Drug repurposing has become an increasingly attractive strategy for accelerating therapeutic innovation, particularly in psychiatry, where novel drug development remains lengthy and costly. Oka N et al. demonstrated that donepezil, an acetylcholinesterase inhibitor, ameliorates the fatigue and depressive symptoms in a subset of patients with post-acute sequelae of SARS-CoV-2 infection. This study illustrates how new applications may be found for established pharmacological agents when guided by mechanistic insights. Complementing these translational efforts, Zhang et al. presented the protocol for a randomized, double-blind clinical trial evaluating the safety and efficacy of adjunctive esketamine combined with different anesthetic strategies during electroconvulsive therapy in patients with major depressive disorder. Beyond assessing a promising therapeutic combination, this study exemplifies the importance of rigorous clinical trial design in optimizing established interventions and accelerating the translation of mechanistic advances into evidence-based clinical practice. In parallel, Qu Y et al. examined dose-dependent adverse events of esketamine in treatment-resistant depression, providing an important evidence-based framework for balancing therapeutic benefits against potential risks.

Collectively, these studies highlight several important shifts currently reshaping the field of neuropsychopharmacology. Firstly, they underscore the transition from symptom-oriented pharmacology toward mechanism-informed precision-based treatments. Secondly, they demonstrate that advances in receptor-specific modulation, neuroimmune regulation, and systems biology are emerging as complementary rather than competing strategies for therapeutic development. Lastly, advances in -omics research and specific biomarker discovery result in unprecedented opportunities to stratify patients and personalize treatment, moving the field towards precision psychiatry.
